# Circadian Regulator-Mediated Molecular Subtypes Depict the Features of Tumor Microenvironment and Indicate Prognosis in Head and Neck Squamous Cell Carcinoma

**DOI:** 10.1155/2023/9946911

**Published:** 2023-06-12

**Authors:** Ling Aye, Zhanying Wang, Fanghua Chen, Yujun Xiong, Jiaying Zhou, Feizhen Wu, Li Hu, Dehui Wang

**Affiliations:** ^1^Department of Otolaryngology, Shanghai Eye & ENT Hospital, Fudan University, Shanghai 200031, China; ^2^Five-Year Program Clinical Medicine, Grade of 2019, West China School of Medicine, Sichuan University, Chengdu, 610041 Sichuan, China; ^3^Key Laboratory of Carcinogenesis and Cancer Invasion of Ministry of Education, Fudan University, Shanghai 200032, China; ^4^Department of Gastroenterology, Beijing Hospital, National Center of Gerontology, Institute of Geriatric Medicine, Chinese Academy of Medical Sciences, Beijing, China; ^5^Department of Otolaryngology, Shanghai General Hospital, Shanghai Jiao Tong University School of Medicine, Shanghai 200080, China; ^6^Laboratory of Epigenetics, Institutes of Biomedical Sciences, Fudan University, Shanghai 200032, China; ^7^Key Laboratory of Birth Defects, Children's Hospital of Fudan University, Shanghai 201102, China

## Abstract

**Introduction:**

Circadian rhythm is involved in multiple biological activities and implicated in cancer development. However, the role of circadian rhythm in head and neck squamous cell carcinoma (HNSCC) has not been fully interpreted yet. Herein, the present study set out to explore the significance of circadian regulator genes (CRGs) in HNSCC.

**Materials and Methods:**

The molecular landscape and clinical significance of 13 CRGs in HNSCC were explored based on The Cancer Genome Atlas (TCGA). The biological functions of PER3, a key CRG, were validated by cellular experiments. The correlation of CRGs with microenvironment, pathway activities, and prognosis was determined by bioinformatic algorithms. A novel circadian score was introduced to evaluate the circadian modification pattern of each patient and further validated in an independent cohort from the Gene Expression Omnibus (GEO) dataset.

**Results:**

CRGs presented high heterogeneity in HNSCC at both genomic and transcriptomic levels. Specifically, PER3 indicated a better prognosis and inhibited HNSCC cell proliferation. Moreover, HNSCC tissues displayed three circadian regulator patterns with distinct clinical outcomes, transcriptomic characteristics, and microenvironment features. Circadian score was an independent risk factor and exhibited excellent predictive efficiency in both the training cohort from the TCGA database and the validation cohort from the GEO database.

**Conclusions:**

CRGs played an indispensable role in HNSCC development. An in-depth exploration of circadian rhythm would improve the understanding of HNSCC carcinogenesis and confer novel insights for future clinical practices.

## 1. Introduction

Head and neck squamous cell carcinoma (HNSCC) currently stands as the sixth most common cancer worldwide [1, 2], representing approximately 6% of all new cancer cases [3]. Despite the inspiring efficacy of surgery, chemotherapy, radiotherapy, and immunotherapy in early diagnosed cases, the clinical outcomes and quality of life of HNSCC patients with advanced HNSCC are still far from satisfaction, which is largely due to the inadequate understanding of HNSCC pathogenesis [4–6].

The recent advent of integrative multiomics technologies has manifested the importance of specific molecular pathways in carcinogenesis, among which circadian rhythm was deemed a promising target for cancer therapy [7]. Circadian rhythm refers to the periodic changes of organisms over time, including behavioral patterns and morphological structures, which plays an important role in a variety of physiological and biochemical processes [8]. The material basis of this rhythm is the molecular chronometer, also known as the circadian clock [9]. The circadian clock is regulated by a cell-autonomous and self-sustained oscillation mechanism, which is consisted of three elements: input pathways for entrainment, the pivot oscillator generating the rhythmicity, and output pathways for controlling rhythmic behaviors [10]. The input pathways, usually composed of external environmental factors such as light and temperature, are processed by oscillators to maintain the 24-hour cycle of an organism and ultimately affect the output pathways, such as body temperature and heart rate [11]. The oscillator is essentially a transcription-translation feedback loop (TTFL) [12]. In the canonical model for the mammalian circadian clock, CLOCK-BMAL1 complex activates the transcription of Cryptochrome (CRY) and Period (PER) genes by binding to their promoters during the day [11]. The resulting protein, reversely, dissociates the combination of CLOCK-BMAL1 and inhibits CLOCK-BMAL1-mediated transcriptional activation during the night [13, 14]. Other molecules, such as basic helix-loop-helix-PER-ARNT-SIM (bHLH-PAS) [15], casein kinase 1 (CSNK1) [16], and REV-ERB*α*/*β* (NR1D) [17], also participate in the composition of autonomous feedback loops, respectively.

The oscillator is conserved across species and exists in most body cells [18], allowing them to manipulate various biological processes and, therefore, participate in numerous diseases, including diabetes [19], cardiovascular diseases [20], and cancers [21]. Particularly, the disorder of circadian rhythm-related genes (CRGs) has been observed in many types of cancers, including breast cancer [22], lung cancer [23], and glioma [24]. As central regulators of multiple biological processes, CRGs can directly alter cancer cell behaviors by coordinating oncogenic or tumor-suppressor signaling pathways. For example, in vivo screening of key transcription factors shows that CLOCK and BMAL1 are potent regulators of stemness-related pathways and required for the proliferation of acute myeloid leukemia cells [25]. On the other hand, CRGs are indirectly implicated in cancer development by affecting other factors like tumor microenvironment (TME) [26]. CLOCK has been documented to recruit immune-suppressive microglia by directly regulating OLFML3 secretion, ultimately leading to glioblastoma progression [27]. These studies jointly suggest that circadian rhythm plays a critical role in cancer development and progression. However, the molecular landscape and clinical significance of CRGs in HNSCC remain ambiguous, which hamper the understanding of the HNSCC pathogenesis and the development of effective therapies.

Herein, we integrated the genome and transcriptome information of head and neck squamous cell carcinoma (HNSCC) based on The Cancer Genome Atlas (TCGA) to comprehensively evaluate possible circadian regulator patterns (CRPs) in HNSCC. The interplay of CRPs with different TME and signal pathways was determined using novel bioinformatic algorithms. Moreover, we identified 341 differentially expressed genes (DEGs) from three CRPs, among which 67 DEGs were correlated with overall survival (OS) and termed circadian signature genes (CSGs). Through combing machine learning methods, we established and validated a circadian score system based on CSGs to describe the circadian rhythm patterns of individual HNSCC patient. Our results demonstrated that circadian score might serve as a surrogate biomarker for indicating clinical outcomes of HNSCC patients.

## 2. Materials and Methods

### 2.1. Collection of HNSCC Datasets and Preprocessing

The main concept and workflow of our study are shown in Supplementary Figure 1. The genetic alteration, mRNA expression profile, and corresponding clinical information of the TCGA cohort were downloaded from the Cancer Genomics Browser of University of California Santa Cruz (UCSC). The expression profile and corresponding clinical information of GSE65858 were downloaded from the Gene Expression Omnibus (GEO) database [28]. The clinical characteristics of these patients are summarized in Table 1.

### 2.2. Unsupervised Clustering for CRGs

The mRNA sequencing data were transformed into transcripts per kilobase million (TPM) values and normalized for cluster analysis. Unsupervised clustering methods were used to identify different circadian rhythm-related subtypes by using the package ConsensusClusterPlus. A total of 13 CRGs (CSNK1E, PER1, RHYTHM, CRY1, CRY2, BHLHE41, PER2, PER3, ARNTL, NPAS2, BHLHE40, CSNK1D, and NR1D1) were selected based on KEGG_CIRCADIAN_RHYTHM_MAMMAL gene set from Kyoto Encyclopedia of Genes and Genomes (KEGG).

### 2.3. DEG Analysis and Function Enrichment Analysis

DEGs were identified at a cutoff of *p* value < 0.05 with the Limma package [29]. Enrichment analysis was performed by using the clusterProfiler package with default parameters [30]. KEGG [31], gene set enrichment analysis (GSEA) [32], Gene Ontology (GO) enrichment analysis, and gene set variation analysis (GSVA) [33] were employed as well. Specifically, hallmark gene sets were downloaded from the Molecular Signature Database (MSigDB), and immune-related gene sets were downloaded from the study of Mariathasan et al. [34].

### 2.4. Estimation of Immune Cell Infiltration

The immune infiltration was analyzed via ESTIMATE [35], CIBERSORT [36], and single-sample gene set enrichment analysis (ssGSEA) algorithm. The gene sets utilized in ssGSEA were downloaded from the study of Charoentong et al. [37].

### 2.5. Generation of Circadian Score

We determined the prognostic value for DEGs among three CRPs by the univariable Cox proportional hazard regression analysis. The DEGs with significant prognostic value (*p* < 0.05) were termed circadian signature genes (CSGs). Then, the least shrinkage and selection operator (LASSO) and multivariable Cox model were utilized to construct circadian score. The circadian score was constructed using 6 CSGs (DEFB1, MTHFD2, KCTD11, POMP, RPL12, and TNFRSF12A) with a linear combination of their expression values. These inputs were weighted with the regression coefficients from the stepwise Cox regression analyses. The circadian score was shown as follows:

(1) $$\begin{array}{c} \text{Circadian score}=\overset{6}{\underset{n=1}{\sum }}\left(\text{coefficient}\left(\text{gene i}\right)*\text{expression }\left(\text{gene i}\right)\right). \end{array}$$

### 2.6. Cell Culture and Functional Assays

The HNSCC cell lines 6-10B and Tu 686 were purchased from FuHeng Cell Center (Shanghai, China). The cell lines mentioned above were cultured in complete medium: RPMI Medium 1640 (Gibco, USA) supplemented with 10% fetal calf serum (Gibco, USA), penicillin (100 units/ml) and streptomycin (100 *μ*g/ml) at 37°C in a thermostatic incubator with 5% CO_2_. All cells were validated using DNA short tandem repeat analysis each six months and tested for mycoplasma contamination using One-Step Mycoplasma Detection Kit (Yeason, Shanghai, China) each four weeks.

Functional assays were conducted in two groups: a negative group (sh-NC) and an shRNA knock-down group (sh-PER3). Cell proliferation was detected by the Cell Counting Kit-8 (CCK-8) and clone formation assays. In brief, cells were inoculated into 96-well plates (1000 cells/well). At each time point (1st, 2nd, 3rd, 4th, and 5th day), 10 *μ*l of CCK-8 solution (Yeason, Shanghai, China) was added to the sextuplicate wells. The plates were incubated for 1.5 h, and the optical density values were detected. For clone formation assays, cells were seeded in a six-well plate (1000 cells/well) with the culture medium refreshed every 3 days for 2 weeks. Following the 2 weeks, the cells were washed with PBS, fixed, stained, and counted.

### 2.7. Virus Transfection

The vectors of plko-Puromycin-EGFP-shRNA-PER3 and plko-Puromycin-EGFP-NC were purchased from Ruoji Technology (Shanghai, China) and were transfected into 6-10B and Tu 686 cells. The sequences of short-hairpin RNA (shRNA) were as follows:

shRNA1: CCCUCGGAGAGACGCAAUAAA (F) and UUUAUUGCGUCUCUCCGAGGG (R)

shRNA2: AUGACCAUGAAGUUAUCAUUG (F) and CAAUGAUAACUUCAUGGUCAU (R)

shRNA3: CGACAGCCUCUUCUGCGAUAU (F) and AUAUCGCAGAAGAGGCUGUCG (R)

These three target sequences were mixed with the same proportion. After transfection, the cells with suitable fluorescence expression were screened with puromycin at a concentration of 4 *μ*g/ml.

### 2.8. Real-Time Polymerase Chain Reaction (Real-Time PCR)

Total RNA was purified using Mini BEST Universal RNA extraction KIT (TaKaRa, Japan), and cDNA was synthesized using the Prime-Script RT Master Mix (TaKaRa, Japan) according to the manufacturer's instructions. Real-time PCR was performed using SYBR Green Realtime PCR Master Mix (Yeasen, China). Samples from each experiment were analyzed in triplicate. The primer sequences used in this study were as follows:

GAPDH: GGACTCATGACCACAGTCCA (F) and CCAGTAGAGGCAGGGATGAT (R)

PER3: AGCGTTCAAGCAAACAGTGAG (F) and CAAGCAGGTCAACAAAGTGAGA (R)

### 2.9. In Vivo Assay

Five-week-old athymic BALB/c female nude mice were purchased from the Jihui Laboratory Animal Care Co., Ltd. (Shanghai, China) and maintained in pathogen-free environment, in accordance with the stated guidelines of 3Rs (replacement, reduction, and refinement). These mice were randomly divided into two subgroups (*n* = 3/each group): sh-NC group and sh-PER3 group. A total of 5 × 10^6^ cells were injected into the back of the mice to generate subcutaneous tumors. Tumor volume was measured regularly until the 24th day and calculated using the formula: length × width^2^ × 0.5. Twenty-four days after injection, the tumor specimens were surgically removed, weighted, and fixed. The animal experiments met the requirements of the Animal Ethics Committee of Shanghai Eye & ENT Hospital, Fudan University.

### 2.10. Statistical Analysis

The results were expressed as the mean ± standard deviation. Parameter test or rank-sum test was used for comparisons between groups. The Bonferroni test was used for multiple comparisons. Categorical data were analyzed by the chi-square test or Fisher's exact test. For survival analysis, the Kaplan-Meier method, log-rank test, and Cox regression analysis were used to test the prognostic value. All statistical tests were bilateral with *p* value < 0.05 being statistically significant. R software (4.1.1) and GraphPad Prism 7 were used for data analyses.

## 3. Results and Discussion

### 3.1. The Molecular Landscape of CRGs in HNSCC

**Figure 1:**
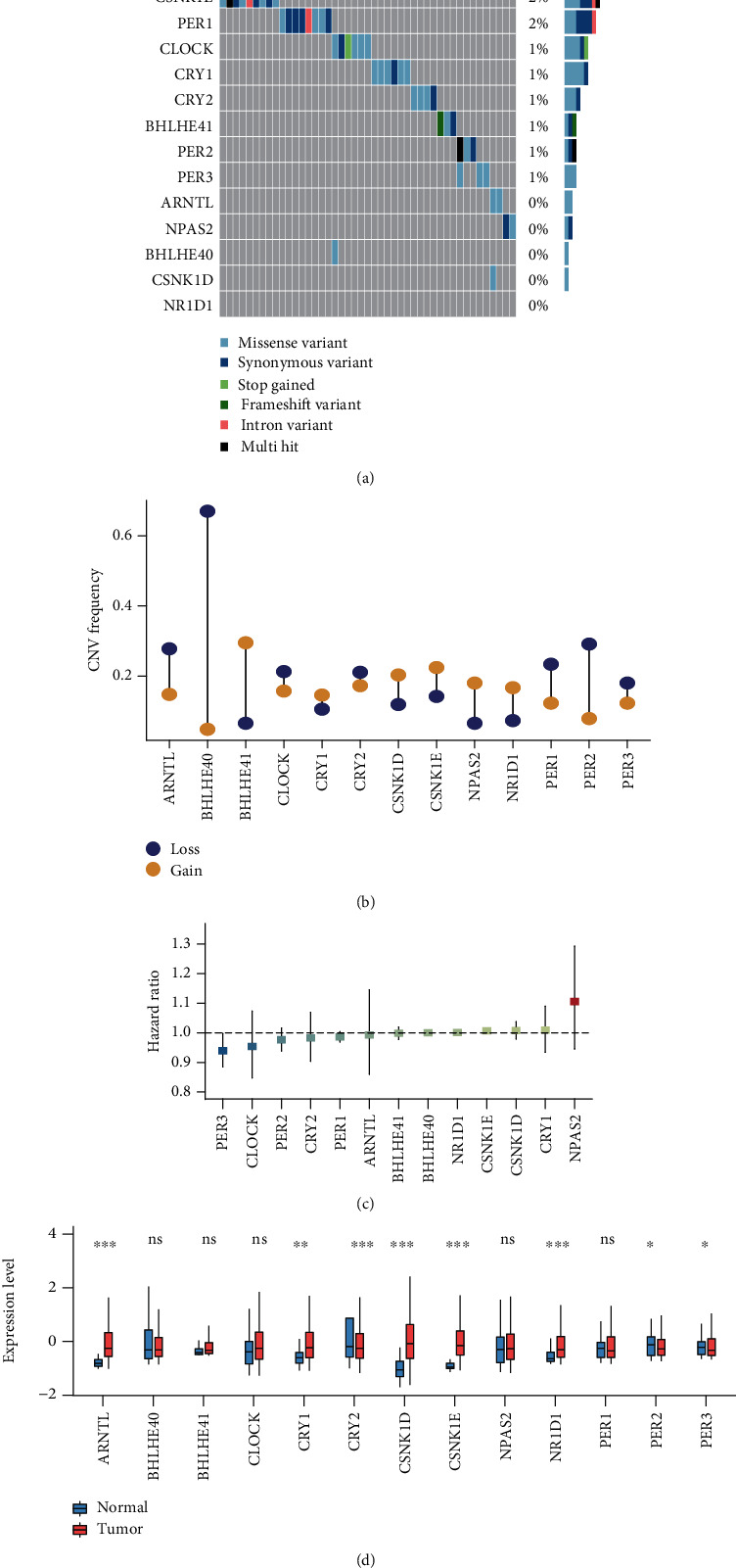
Genetic alteration, expression variation, and clinical significance of CRGs in HNSCC. (a) The mutation frequency of CRGs in TCGA HNSCC cohort. (b) The CNV frequency of CRGs in the TCGA HNSCC cohort. Gain, yellow; loss, blue. (c) The forest plot of the univariate Cox regression model depicting the prognostic value of CRGs in TCGA HNSCC cohort. Hazard ratio > 1: risk factors for survival. Hazard ratio < 1: protective factors for survival. (d) Expression level of CRGs between normal and tumor tissues in the TCGA HNSCC cohort. Ns: not significant; ^∗^: *p* < 0.05; ^∗∗^: *p* < 0.01; ^∗∗∗^: *p* < 0.001.

In this study, we identified 13 CRGs and summarized the incidence of somatic mutations of these CRGs based on the TCGA cohort. Genetic mutations of CRGs occurred in approximately 8.8% of HNSCC patients, among which CSNK1E and PER1 exhibited the highest mutation frequency (Figure 1(a)). The copy number variation (CNV) analysis revealed significant copy number deletions for BHLHE40 and PER2 and copy number amplifications mainly for BHLHE41 (Figure 1(b)). The univariable Cox analysis was used to assess the prognostic value of each CRG (Figure 1(c)). The result showed that the hazard ratio of PER3 was the lowest among 13 CRGs, suggesting its role as a protective factor in HNSCC. In addition, the differential expression patterns of CRGs between HNSCC tumors and adjacent normal tissues were also analyzed. The result manifested that five CRGs were highly expressed in tumors (Figure 1(d)), while opposite trends were observed for CRY2, PER2, and PER3. The aforementioned analyses suggested the high heterogeneity of genetic alteration and expressional landscape of CRGs in HNSCC, implying that dysregulation of CRGs was of great significance in HNSCC.

### 3.2. PER3 Knockdown Promoted Proliferation of HNSCC Cell

**Figure 2:**
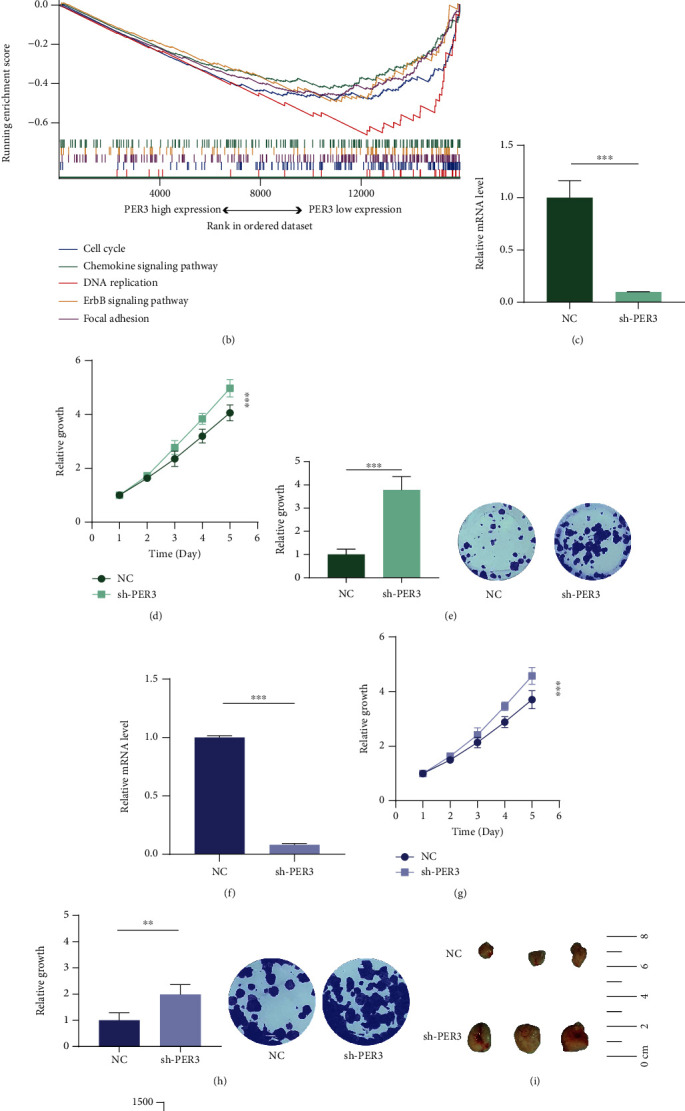
Downregulation of PER3 promoted HNSCC cell proliferation. (a, b) GSEA GO and GSEA KEGG analyses identified proliferation-related pathways enriched in the low PER3 expression samples. (c, f) The efficiency of PER3 transfection in Tu 686 and 6-10B cells was confirmed by real time-PCR. (d, g) Downregulation of PER3 in Tu 686 and 6-10B cells promoted cell proliferation as determined by CCK-8. (e, h) Colony formation assays showed that inhibition of PER3 in Tu 686 and 6-10B cells promoted cell proliferation. (i) Subcutaneous tumors obtained from nude mice implanted with the sh-NC or sh-PER3 cell lines. (j) The tumor volume is shown to be significantly increased in sh-PER3 group compared with that in the sh-NC group. (k) The tumor weight is significantly higher in sh-PER3 group than in the sh-NC group. Data represent means ± SD of three independent experiments. ^∗^: *p* < 0.05; ^∗∗^: *p* < 0.01; ^∗∗∗^: *p* < 0.001.

Considering the significant prognostic value and the distinct expression pattern of PER3, PER3 was selected for deeper investigation. The Wilcoxon test showed that PER3 expression was downregulated in patients with higher T stage (*p* = 0.004) and M stage (*p* = 0.009, supplementary Figure 2A-D). GSEA analysis manifested that the enrichment of proliferation-related pathways, including DNA replication and the ErbB pathway, was significantly increased in samples with low expression of PER3 (*p* < 0.001, Figures 2(a) and 2(b)). Thereafter, cellular experiments were performed to verify the effect of PER3 on the biological behaviors of HNSCC cells. Firstly, shRNA was employed to knock down PER3 expression in two typical HNSCC cell lines, and the transfection efficiency was detected by real-time PCR (Figures 2(c) and 2(f)). CCK-8 assay demonstrated that inhibition of PER3 increased HNSCC cell viability in vitro (Figures 2(d) and 2(g)), which was further confirmed by colony formation assay (Figures 2(e) and 2(h)). Subsequently, a subcutaneous xenograft model was established to validate the function of PER3 in vivo (Figure 2(i)). The tumor weight and volume were obviously higher in the sh-PER3 group than those in the sh-NC group (Figures 2(j) and 2(k)). In summary, these results collectively illustrated that PER3 downregulation correlated with proliferation-related pathways and indeed promoted HNSCC cell vitality.

### 3.3. HNSCC Patients Exhibited Three Circadian Rhythm-Related Subtypes with Distinct Prognosis and TME

**Figure 3:**
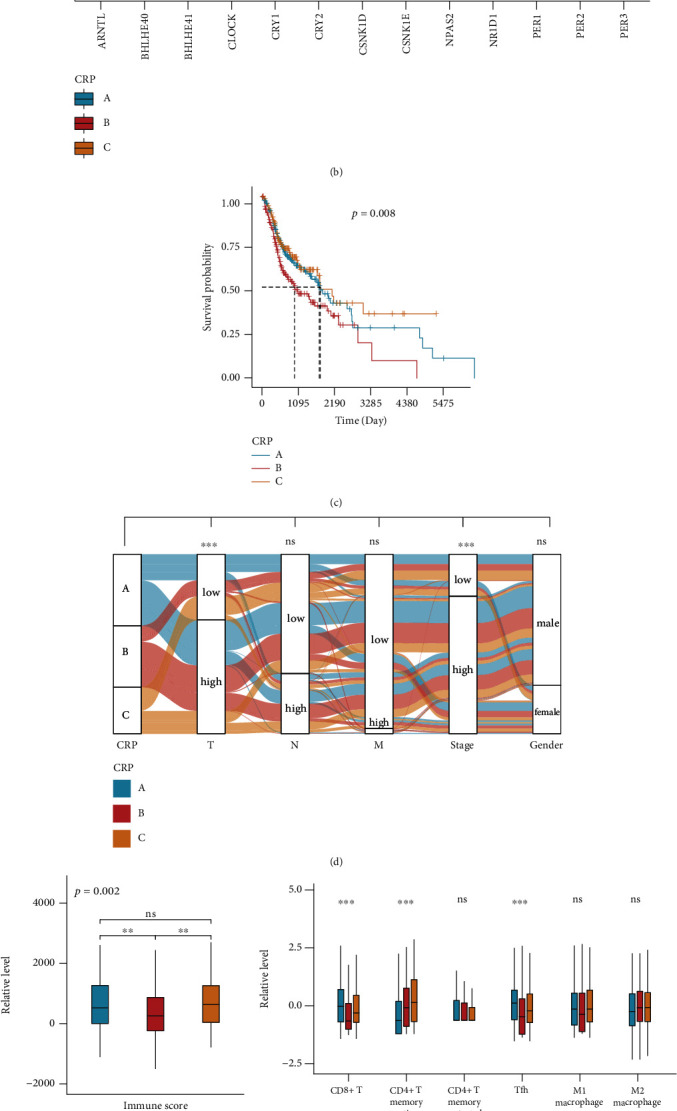
HNSCC patients exhibited three circadian rhythm-related subtypes with distinct prognosis and TME. (a) Consensus clustering matrix of HNSCC patients for *k* = 3. (b) Expression of 13 CRGs in three CRPs. (c) Survival analysis for three CRP subtypes. (d) An alluvial diagram depicting the relationship between CRP and clinical features (T low = T1 + T2; T high = T3 + T4; N low = N0 + N1; N high = N2 + N3 + NX; M low = M0; M high = M1 + MX; grade low = G1 + G2; grade high = G3 + G4 + GX; stage low = I + II; and stage high = III + IV). (e) Boxplots depicted the differences in immune scores in the three clusters. (f, g) Different immune cell component among three CRP subtypes as determined by CIBERSORT and ssGSEA. Ns: not significant; ^∗^: *p* < 0.05; ^∗∗^: *p* < 0.01; ^∗∗∗^: *p* < 0.001.

To explore the expression pattern of CRGs, consensus clustering analysis was conducted based on 13 CRGs to stratify HNSCC patients. Three clusters were achieved and termed CRPs, of which there were 198 cases in CRP-A, 170 cases in CRP-B, and 129 cases in CRP-C (Figure 3(a)). Obviously, CRGs had diverse expression levels among three CRP subtypes (Figure 3(b)). Log-rank analysis revealed that CRP-B presented a particularly prominent survival disadvantage over CRP-A and CRP-C (*p* = 0.008, Figure 3(c)). Chi-square analysis showed that CRP was closely correlated to T stage (*p* < 0.001) and clinical stage (*p* < 0.01, Figure 3(d)). Meanwhile, immune infiltration status was also different in three CRPs, with CRP-B showing the lowest immune score (*p* = 0.002, Figure 3(e)). Specifically, the infiltration of CD8+ T cells, which participated in active antitumor responses and correlated with better prognosis [38], displayed the lowest level in CRP-B (Figure 3(f)). ssGSEA results also validated that CD8+ T cells and cytotoxic T lymphocytes (CTL) were downregulated in CRP-B (Figure 3(g)). Based on these findings, we confirmed that HNSCC samples exhibited three CRPs, characterized by different prognosis and immune infiltration profiles.

### 3.4. Transcriptomic Characteristics of Different CRP Subtypes

**Figure 4:**
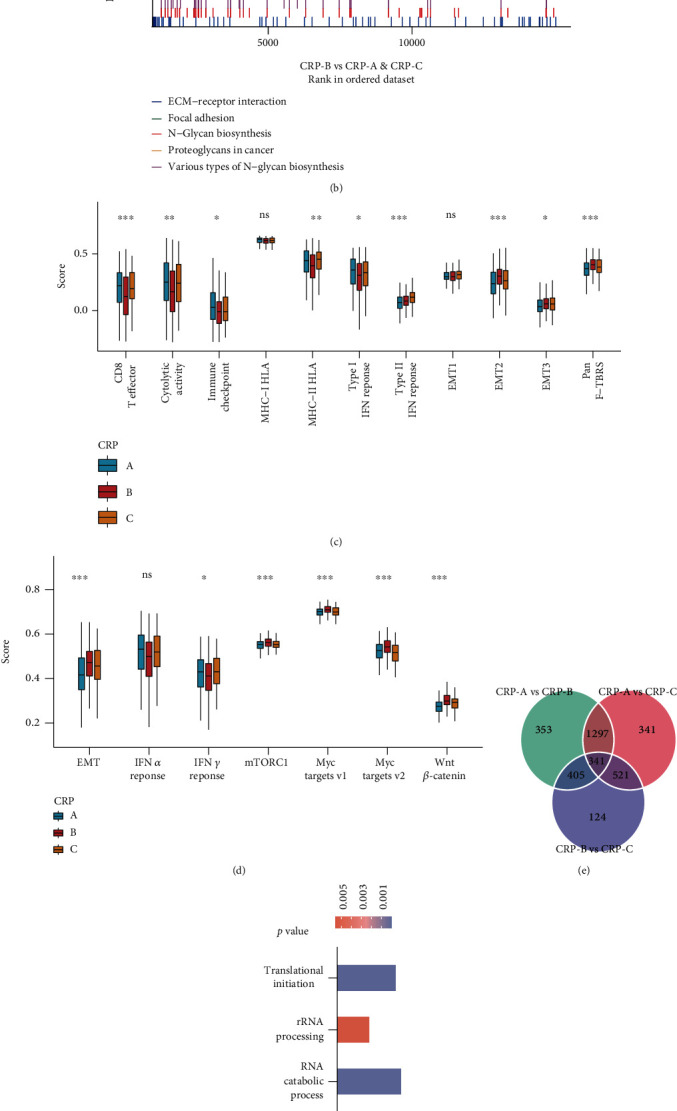
Transcriptomic characteristics of different CRP subtypes. GSEA GO (a) and GSEA KEGG (b) analyses demonstrated stroma- and stemness-related pathways enriched in the CRP-B vs. CRP-A and CRP-C. (c) Boxplot of several immune signatures for three CRPs. (d) GSVA enrichment analysis showing the activation states of possible pathways in different CRPs based on hallmark gene sets. (e) 341 circadian-related DEGs between three CRPs were shown in the Venn diagram. (f) Functional annotation for circadian signature genes using GO enrichment analysis. Ns: not significant; ^∗^: *p* < 0.05; ^∗∗^: *p* < 0.01; ^∗∗∗^: *p* < 0.001.

To elucidate the underlying mechanisms involved in different clinical outcomes and immune contextures among three CRP subtypes, potential pathways were explored using bioinformatic algorithms. GSEA analysis revealed that, compared with CRP-A and CRP-C, CRP-B was primarily enriched in stroma-related processes such as collagen binding and N-glycan biosynthesis (*p* < 0.05, Figures 4(a) and 4(b)). Previous research reported that stromal components could repress antitumor responses in various ways, such as shaping immune cell composition through the formation of geographic barriers and affecting immune cell viability via the release of soluble factors [39]. Therefore, we speculated that stromal components in TME inhibited CD8+ T cell-mediated cytotoxic responses, ultimately leading to the poor prognosis of CRP-B. By combing hallmark gene sets and immune infiltration-related gene sets, we further explored different signal activities by GSVA analysis. The results verified that CRP-B was characterized by the activation of stroma-related pathways, like epithelial-mesenchymal transition (EMT), and the repression of antitumor immune responses, including CD8 effecter, cytolytic activity, and interferon responses (Figures 4(c) and 4(d)). Besides, GSEA and GSVA jointly suggested that stemness-related pathways, such as Wnt and Myc pathways, might be involved in these biological processes (Figures 4(a) and 4(d)).

Differential expression analysis was conducted to further explore the heterogeneity of different CRPs, and 341 DEGs were identified (Figure 4(e)). The univariable Cox analysis revealed that 67 DEGs were associated with overall survival (OS) (*p* < 0.05) and termed CSGs. GO analysis exhibited that CSGs mainly centered around mRNA and protein regulation, in line with the biological essence of circadian clock as a TTFL (Figure 4(f)). To further ascertain the possible role of CSGs, unsupervised clustering analysis was conducted based on the obtained CSGs, which yielded three circadian genomic clusters (CGCs), namely, CGC1-3. Similar to CRP, three CGCs distinguished different clinical characteristics, OS, and immune infiltration level, particularly T cell and CD8+ T cell abundance (supplementary Figure 3). Notably, we found that PER3 expression varied from different CGC subtypes and CRP subtypes (supplementary Figure 2E and F), implying that PER3 might be implicated in different clinical outcomes of these subtypes. Altogether, these results depicted the different transcriptomic features among CRPs, which might be involved in the different TME landscape and clinical outcomes.

### 3.5. Construction of Circadian Score and Exploration of Its Clinical Relevance

**Figure 5:**
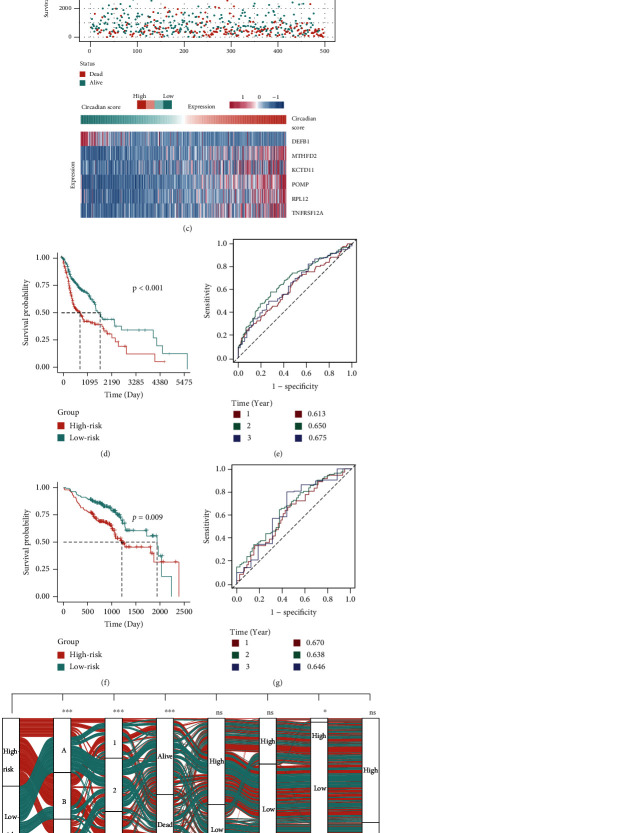
Construction of the circadian score and exploration of its clinical relevance. (a) LASSO coefficient profiles of the 67 genes selected. The dotted vertical line was drawn at the optimal scores by minimum criteria. (b) Six genes were selected using LASSO Cox regression analysis. The dotted vertical line was drawn at the optimal scores by minimum criteria. (c) The risk scores for 497 patients of the TCGA cohort, the survival of each patient, and gene expression distribution in the TCGA cohort. (d) Kaplan-Meier curves of the OS in the TCGA cohort on the basis of the circadian score. (e) ROC analysis of the circadian score for prediction of survival at 1, 2, and 3 years in the TCGA cohort. (f) Kaplan-Meier curves of the OS in the GEO cohort on the basis of the circadian score. (g) ROC analysis of the circadian score for prediction of survival at 1, 2, and 3 years in the GEO cohort. (h) An alluvial diagram showing the relationship of circadian score with clinical characteristics, CRP, and CGC (T low = T1 + T2; T high = T3 + T4; N low = N0 + N1; N high = N2 + N3 + NX; M low = M0; M high = M1 + MX; grade low = G1 + G2; grade high = G3 + G4 + GX; stage low = I + II; and stage high = III + IV). (i) Violin plot of the circadian score for three CRPs. (j) Violin plot of the circadian score for three CGCs. Ns: not significant; ^∗^: *p* < 0.05; ^∗∗^: *p* < 0.01; ^∗∗∗^: *p* < 0.001.

Although our aforementioned findings elaborated on the association among CRPs, prognosis, pathway activities, and TME, these analyses were mainly based on the patient population and could not provide a specific circadian rhythm pattern for an individual patient. Therefore, a circadian score system based on CSGs was introduced. To optimize this model, mainly to minimize multicollinearity, LASSO analysis was utilized with six CSGs ultimately selected for the construction of circadian score (Figures 5(a) and 5(b)). Patients were arranged into high-risk or low-risk groups according to the median cutoff value of circadian score (Figure 5(c)). Log-rank analysis revealed that the OS in the high-risk group was significantly shorter than that in the low-risk group (*p* < 0.001, Figure 5(d)). The 1-, 2-, and 3-year AUC values of the circadian score were 0.613, 0.650, and 0. 675, respectively (Figure 5(e)). Furthermore, the reliability of circadian score was validated in different clinical subgroups and a validation cohort. Subgroup analysis indicated that circadian score was significantly correlated with poor OS in all subgroups except the M-high subgroup (supplementary Figure 4), which might be attributed to the limited sample size of the M-high subgroup (15 out of 497 patients, Table 1). Circadian score also demonstrated extraordinary performance in another independent HNSCC cohort from the GEO database (Figures 5(f) and 5(g)). The correlation of circadian score with clinicopathological parameters, CRP, and CGC was also explored to clarify the features of circadian score. Chi-square analysis displayed that the circadian risk group was significantly correlated with M stage, survival status, CRP, and CGC (Figure 5(h)). The Kruskal-Wallis test revealed that patients in CRP-B showed the highest circadian score (Figure 5(i)), consistent with their worst OS. For CGC, CGC-2 had the lowest circadian score in contrast to other CGC subtypes (Figure 5(j)). The above results jointly revealed that circadian score was an effective biomarker in predicting HNSCC prognosis.

### 3.6. Establishment of a Circadian Score-Based Nomogram

**Figure 6:**
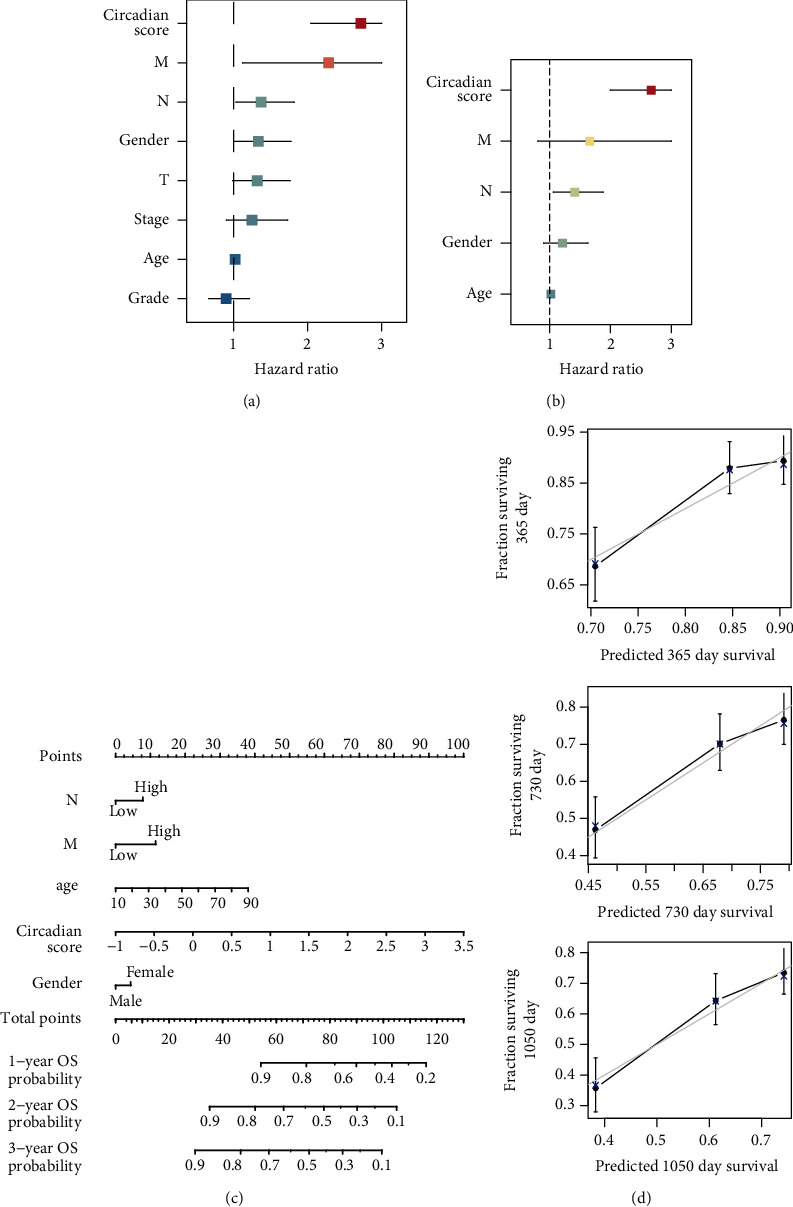
Establishment of a circadian score-based nomogram. (a) Forest plot of the univariable Cox regression analysis. (b) Result of the multivariable Cox regression analysis. (c) A novel nomogram based on gender, age, N, M, and circadian score (T low = T1 + T2; T high = T3 + T4; N low = N0 + N1; N high = N2 + N3 + NX; M low = M0; M high = M1 + MX; grade low = G1 + G2; grade high = G3 + G4 + GX; stage low = I + II; and stage high = III + IV). (d) Calibration curve evaluating the predictive accuracy of the nomogram.

The univariable and multivariable Cox regression analyses were performed to assess the independence of circadian score. The univariable Cox regression analysis demonstrated that age (*p* < 0.01), gender (*p* < 0.05), N (*p* < 0.05), M (*p* < 0.05), and circadian score (*p* < 0.001) were associated with the OS of HNSCC (Figure 6(a)). The multivariable Cox regression analysis revealed that the circadian score was still significantly correlated to OS (*p* < 0.001) and an independent prognostic factor (Figure 6(b)). To facilitate the application of our signature in future clinical practice, a nomogram was built based on the results of the univariable Cox analysis (Figure 6(c)). Besides, calibration curves showed an excellent consistency between the actual and nomogram-predicted probabilities of 1-year, 2-year, and 3-year OS (Figure 6(d)). Together, these results emphasized the clinical significance of the circadian score and the accuracy of the novel circadian score-based nomogram.

## 4. Discussion

Mounting evidence has reported the critical role of circadian clock in mediating immunity, inflammation, and tumorigenesis [40, 41]. However, the influence of CRGs in HNSCC has not been comprehensively delineated.

**Figure 7:**
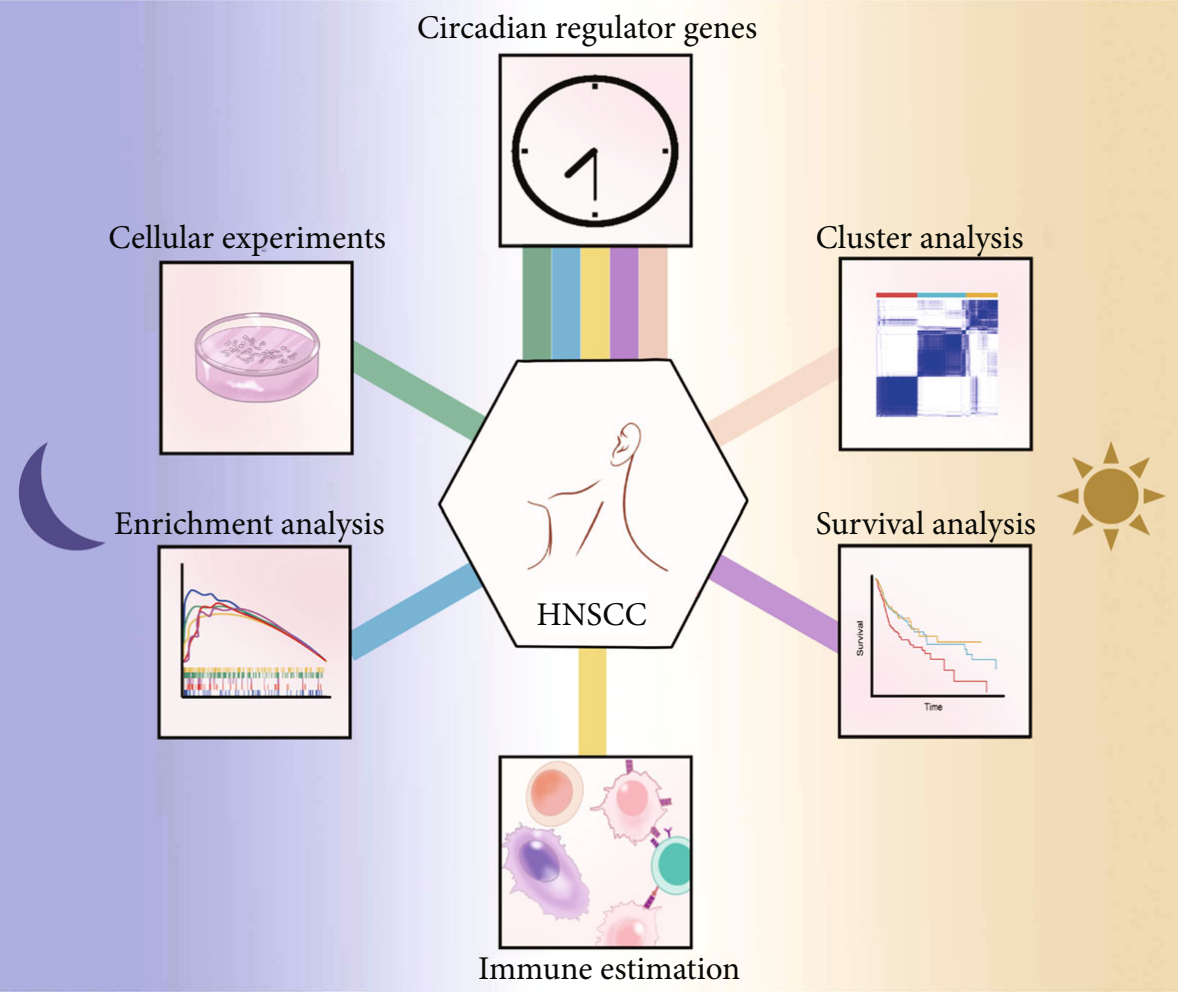
A work scheme illustrating the main content of our study.

Here, we systematically evaluated the role of CRGs in determining different clinical outcomes, pathway activities, and TME features in HNSCC (Figure 7). Specifically, PER3 expression was significantly correlated to a better prognosis among 13 CRGs. Gene enrichment analysis indicated that proliferation-related pathways were enriched in the PER3-low expression samples, suggesting the potential of PER3 in inhibiting cancer cell proliferation. By combining cellular experiments, we found that PER3 depletion could promote HNSCC cell proliferation, consistent with its prognostic value.

Based on 13 CRGs, this study identified three distinct CRPs in HNSCC. Compared to the other two subtypes, samples in the CRP-B subtype exhibited immunosuppressive microenvironment and had the worst OS rate, in line with the critical role of immune surveillance in HNSCC progression [42]. In addition, gene enrichment analysis displayed that CRP-B shared a feature of stroma- and stemness-related pathways, such as EMT and Wnt pathways. It is widely recognized that cancer cells maintain a dynamic interaction with their surrounding stromal components to affect the local immune contexture mutually. For instance, Evan and Vousden [43] discovered that mesothelial cell-derived fibroblasts could induce naive CD4+ T cells into regulatory T cells. Meanwhile, stemness is reported to be associated with immunologically “cold” tumors. Specifically, Wnt signaling pathways can dampen the functions of dendritic cells and effector T cells via secretion of IL-10 and IL-12, leading to an immunosuppressive microenvironment [44, 45]. Consistent with these previous findings, we speculated that activation of stemness- and stroma-related pathways caused an immunosuppressive TME, eventually resulting in the worst prognosis of CRP-B.

Furthermore, 341 DEGs were identified among three CRPs. The univariable Cox regression analysis revealed that 67 out of 341 DEGs were correlated with the OS and termed CSGs. GO analysis indicated that CSGs revolved around transcription and translation regulation, highlighting the central role of the circadian clock in mediating various biological activities. Based on CSGs, three CGCs were identified and found to be closely correlated to TME and OS. These results collectively unraveled that three circadian-related subtypes did exist in HNSCC, in which CRGs and CSGs were vital for the clinical outcomes of HNSCC by shaping TME.

The complexity of cancer has long constituted a main obstacle to precision medicine [46]. To overcome heterogenicity and provide accurate prediction, we innovatively established a six CSG-based circadian score system by performing LASSO and Cox regression analyses. Patients with higher risk scores tended to have shorter OS than those with lower risk scores in both the training cohort from the TCGA dataset and the validation cohort from the GEO dataset. Moreover, we constructed a quantitative nomogram by integrating the circadian score and other clinical features, which exhibited extraordinary reliability as examined by calibration plots. Notably, these six CSGs assume essential roles in numerous human cancers and have been reported as crucial regulators of various immune responses. For instance, MTHFD2 can induce immune escape of cancer cells by promoting the expression of basal and IFN-*γ*-stimulated PD-L1 [47]. Inhibition of POMP can result in the accumulation of cyclin-dependent kinase inhibitors and thus hamper cancer cell proliferation in vitro and in vivo [48].

However, our study still has some limitations. Since this is a retrospective analysis, there may be unavoidable bias in our results. Future prospective studies are warranted to validate the reliability of our conclusion. On the other hand, although this study has depicted the relationship between circadian rhythm and TME, the specific mechanisms remain to be further investigated.

Evidence has reported the critical role of circadian clock in mediating immunity, inflammation, and tumorigenesis [40, 41]. However, the influence of CRGs in HNSCC has not been comprehensively delineated.

Here, we systematically evaluated the molecular landscape and prognostic value of 13 CRGs and discovered that PER3 expression was significantly correlated to a better prognosis (Figure 7). Gene enrichment analysis indicated that proliferation-related pathways were enriched in the PER3-low expression samples, suggesting the potential of PER3 in inhibiting cancer cell proliferation. By combining cellular experiments, we found that PER3 depletion could promote HNSCC cell proliferation, consistent with its prognostic value.

## 5. Conclusions

In conclusion, this study investigated the molecular landscape and clinical significance of CRGs in HNSCC. By mediating various pathways and shaping distinct TME, CRGs play an indispensable role in the heterogeneity of HNSCC. A comprehensive investigation of circadian rhythm of individual patient would contribute to the understanding of cancer development and guide more personalized treatment strategies.

## Figures and Tables

**Table 1:** Clinical characteristics of enrolled groups.

Characteristics	Levels	TCGA cohort	GEO cohort
*n*		497	270

Age (year)	Mean (SD)	61.12 (11.93)	60.12 (10.34)

Survival time (day)	Mean (SD)	913.82 (884.26)	883.03 (451.72)

Gender (%)	Female	134 (27.0)	47 (17.4)
	Male	363 (73.0)	223 (82.6)

Stage (%)	I	20 (4.0)	18 (6.7)
	II	96 (19.3)	37 (13.7)
	III	109 (21.9)	37 (13.7)
	IV	272 (54.7)	178 (65.9)

T (%)	T1	35 (7.0)	35 (13.0)
	T2	147 (29.6)	80 (29.6)
	T3	135 (27.2)	58 (21.5)
	T4	180 (36.2)	97 (35.9)

N (%)	N0	245 (48.9)	94 (34.8)
	N1	85 (17.0)	32 (11.9)
	N2	160 (31.9)	132 (48.9)
	N3	7 (1.4)	12 (4.4)
	NX	4 (0.8)	0 (0.0)

M (%)	M0	482 (97.0)	263 (97.4)
	M1	5 (1.0)	7 (2.6)
	MX	10 (2.0)	0 (0.0)

Grade (%)	NA	3 (0.6)	—
	G1	62 (12.4)	—
	G2	299 (59.7)	—
	G3	119 (23.8)	—
	G4	2 (0.4)	—
	GX	16 (3.2)	—

Survival status (%)	Alive	279 (56.1)	176 (65.2)
	Dead	218 (43.9)	94 (34.8)

## Data Availability

Genomic and transcriptomic data and patients' clinical data are available at UCSC Xena (https://xena.ucsc.edu/) and GEO database (https://www.ncbi.nlm.nih.gov/geo/).

## Abbreviations

AUC: Area under curve
CCK-8: Cell Counting Kit-8
CGC: Circadian genomic cluster
CNV: Copy number variation
CRG: Circadian regulator genes
CRP: Circadian regulator pattern
CRY: Cryptochrome
CSG: Circadian signature genes
CTL: Cytotoxic T lymphocytes
DEG: Differentially expressed gene
EMT: Epithelial-mesenchymal transition
GEO: Gene Expression Omnibus
GO: Gene Ontology
GSEA: Gene set enrichment analysis
GSVA: Gene set variation analysis
HNSCC: Head and neck squamous cell carcinoma
KEGG: Kyoto Encyclopedia of Genes and Genomes
LASSO: Least shrinkage and selection operator
NC: Negative control
OS: Overall survival
PER: Period
real-time PCR: Real-time polymerase chain reaction
ROC: Receiver operating characteristic
shRNA: Short-hairpin RNA
ssGSEA: Single sample GSEA
TCGA: The Cancer Genome Atlas
TME: Tumor microenvironment
TPM: Transcripts per kilobase million
TTFL: Transcription-translation feedback loop
UCSC: California Santa Cruz.

## Supplementary Materials

Supplementary Materials Figure 1: workflow of our study. The workflow of our study and analytical pipeline. Figure 2: comparison of PER3 expression in different subgroups. Box plots of PER3 expression in different T (A), N (B), M (C), stage (D), CRP subtypes (E), and CGC subtypes (F). Figure 3: CGC exhibited different TME and prognosis. (A) Consensus clustering matrix of HNSCC patients based on CSGs (*k* = 3). (B) Expression level of CSGs in the TCGA cohort. (C) A Sankey diagram exhibiting the relationship of CGCs, CRP and clinical features (T low = T1 + T2; T high = T3 + T4; N low = N0 + N1; N high = N2 + N3 + NX; M low = M0; M high = M1 + MX; grade low = G1 + G2; grade high = G3 + G4 + GX; stage low = I + II; and stage high = III + IV). (D) OS of different CGC subtypes. (E) A boxplot of CD8+ T cell abundance for three CGCs. (F) Difference in immune signatures among different CGCs. (G) GSVA analysis among different CGCs. Figure 4: validation of the performance of circadian score in different subgroups. Kaplan-Meier curves of OS based on circadian score in different T (A), N (B), M (C), stage (D), grade (E), and gender (F).
