# Supplementary figures and images for: Circadian Regulator-Mediated Molecular Subtypes Depict the Features of Tumor Microenvironment and Indicate Prognosis in Head and Neck Squamous Cell Carcinoma
Source: J Immunol Res. 2023 Jun 12;2023:9946911. doi: 10.1155/2023/9946911 (PMC10279500; doi:10.1155/2023/9946911)

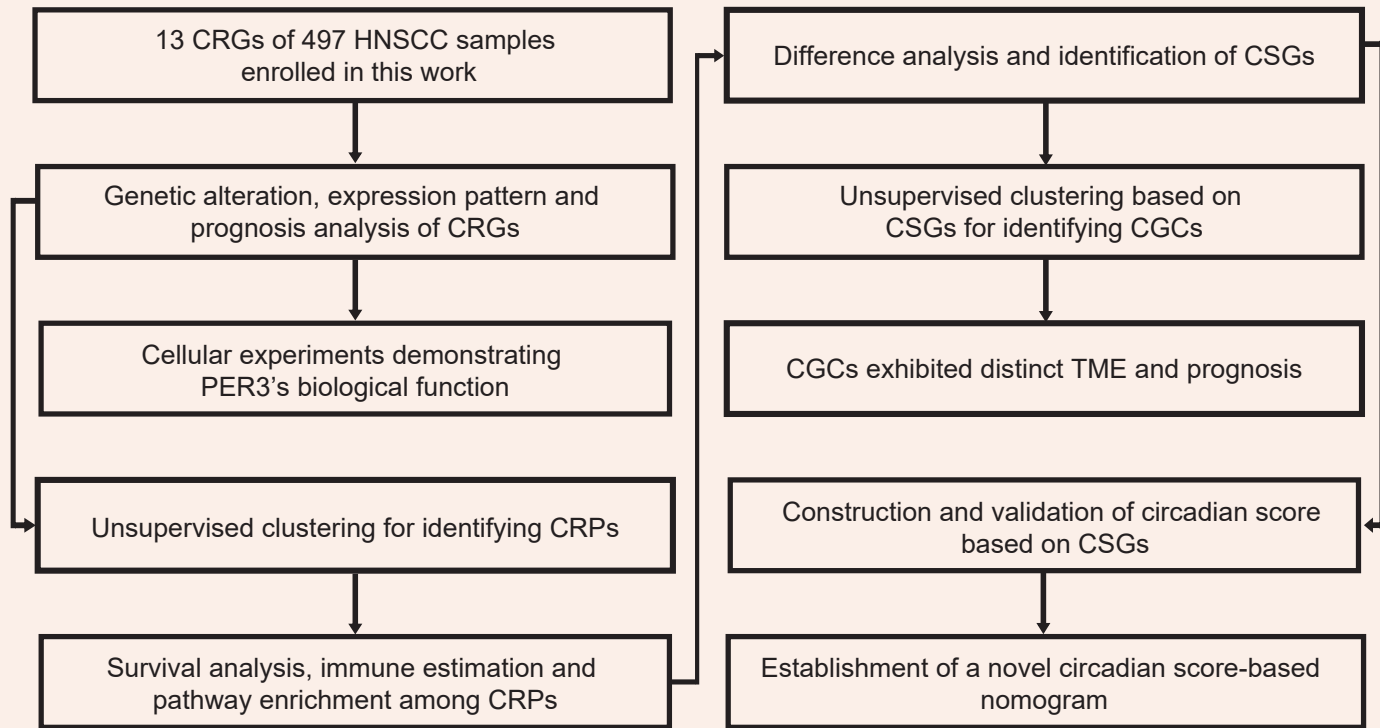

Supplement: Supplementary Materials — Figure 1: workflow of our study. The workflow of our study and analytical pipeline. Figure 2: comparison of PER3 expression in different subgroups. Box plots of PER3 expression in different T (A), N (B), M (C), stage (D), CRP subtypes (E), and CGC subtypes (F). Figure 3: CGC exhibited different TME and prognosis. (A) Consensus clustering matrix of HNSCC patients based on CSGs (k = 3). (B) Expression level of CSGs in the TCGA cohort. (C) A Sankey diagram exhibiting the relationship of CGCs, CRP and clinical features (T low = T1 + T2; T high = T3 + T4; N low = N0 + N1; N high = N2 + N3 + NX; M low = M0; M high = M1 + MX; grade low = G1 + G2; grade high = G3 + G4 + GX; stage low = I + II; and stage high = III + IV). (D) OS of different CGC subtypes. (E) A boxplot of CD8+ T cell abundance for three CGCs. (F) Difference in immune signatures among different CGCs. (G) GSVA analysis among different CGCs. Figure 4: validation of the performance of circadian score in different subgroups. Kaplan-Meier curves of OS based on circadian score in different T (A), N (B), M (C), stage (D), grade (E), and gender (F). [file 9946911.f1.zip › Sfigure1.pdf]

**A**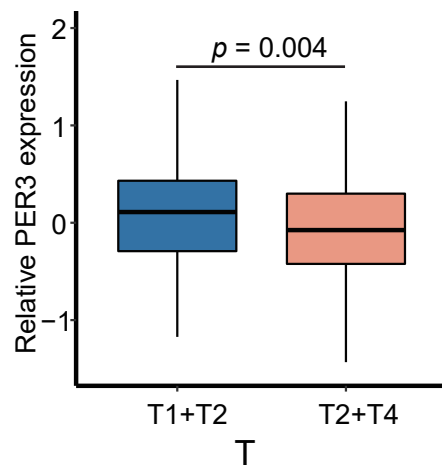**B**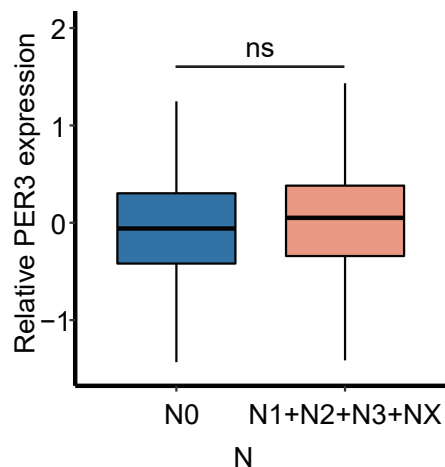**C**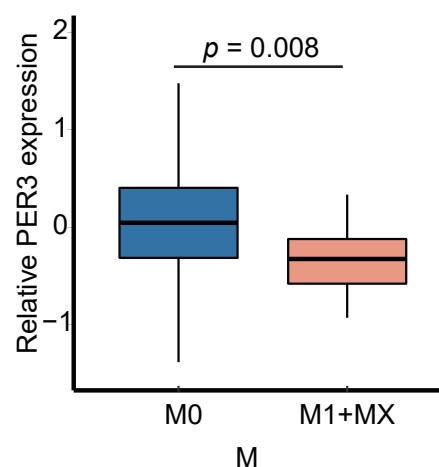**D**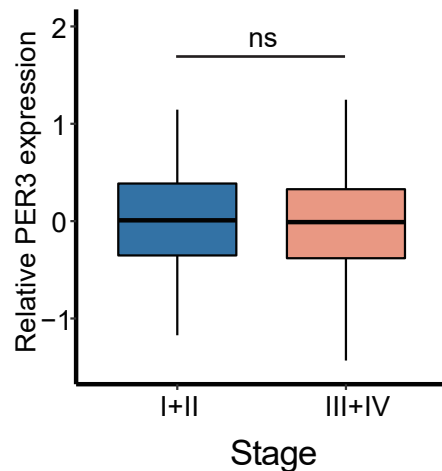**E**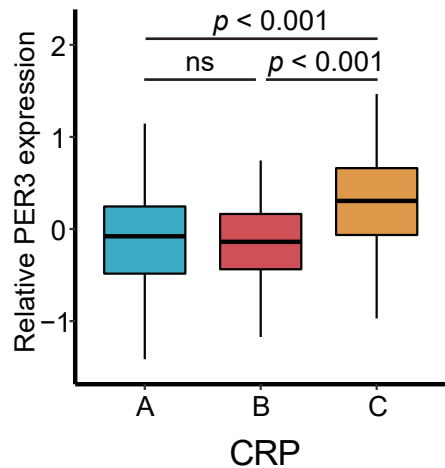**F**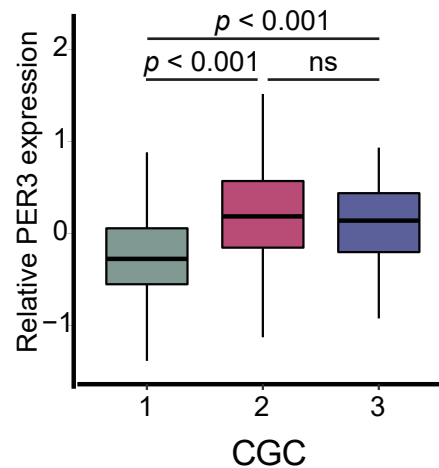

Supplement: Supplementary Materials — Figure 1: workflow of our study. The workflow of our study and analytical pipeline. Figure 2: comparison of PER3 expression in different subgroups. Box plots of PER3 expression in different T (A), N (B), M (C), stage (D), CRP subtypes (E), and CGC subtypes (F). Figure 3: CGC exhibited different TME and prognosis. (A) Consensus clustering matrix of HNSCC patients based on CSGs (k = 3). (B) Expression level of CSGs in the TCGA cohort. (C) A Sankey diagram exhibiting the relationship of CGCs, CRP and clinical features (T low = T1 + T2; T high = T3 + T4; N low = N0 + N1; N high = N2 + N3 + NX; M low = M0; M high = M1 + MX; grade low = G1 + G2; grade high = G3 + G4 + GX; stage low = I + II; and stage high = III + IV). (D) OS of different CGC subtypes. (E) A boxplot of CD8+ T cell abundance for three CGCs. (F) Difference in immune signatures among different CGCs. (G) GSVA analysis among different CGCs. Figure 4: validation of the performance of circadian score in different subgroups. Kaplan-Meier curves of OS based on circadian score in different T (A), N (B), M (C), stage (D), grade (E), and gender (F). [file 9946911.f1.zip › Sfigure2.pdf]

A

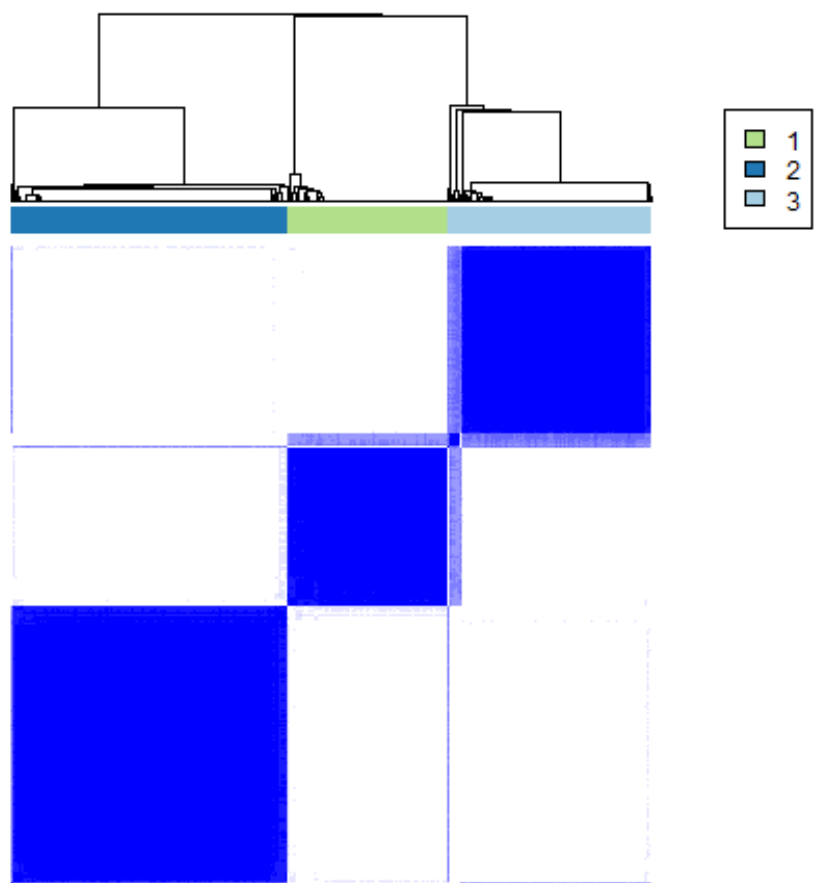

B

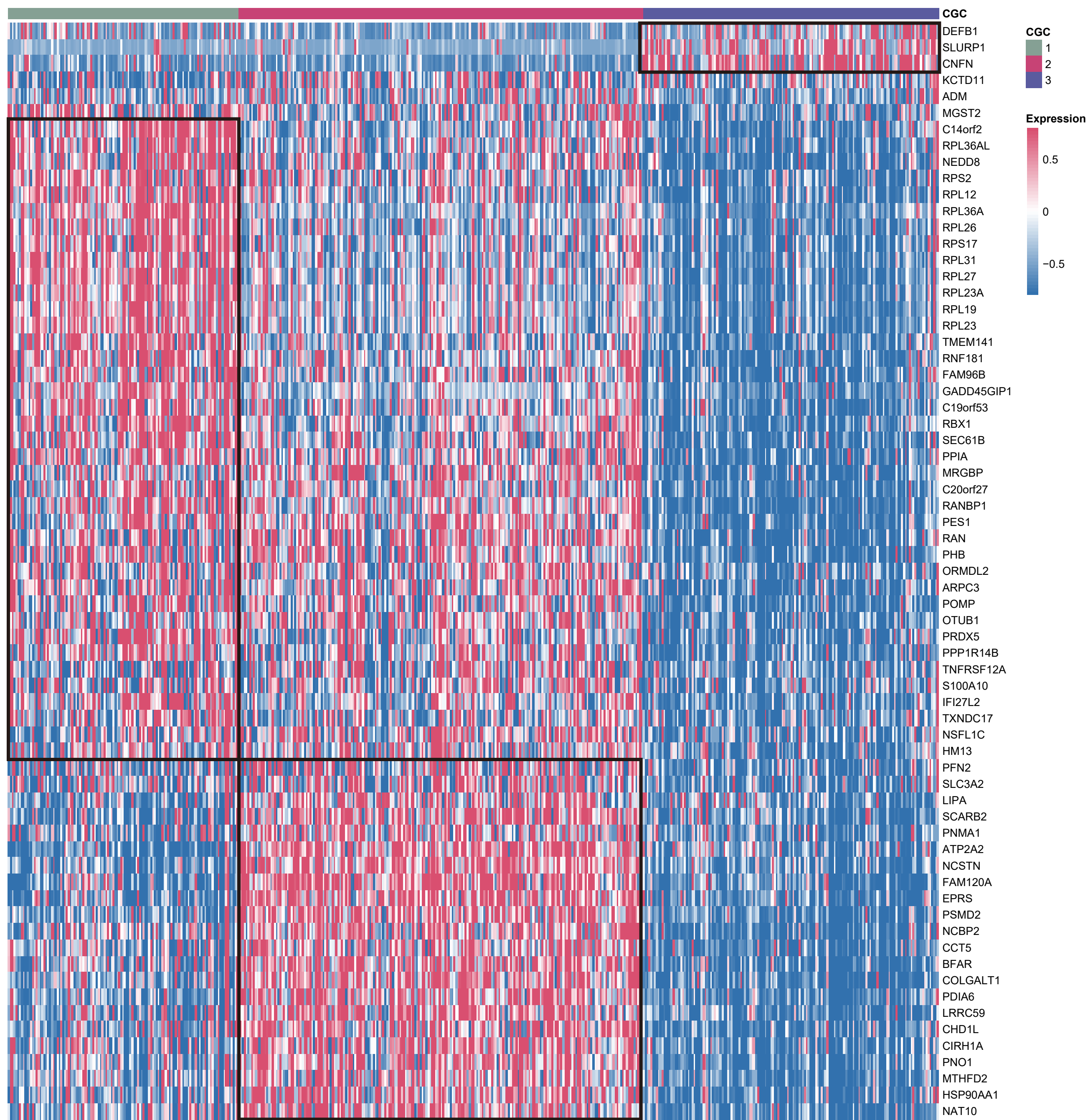

C

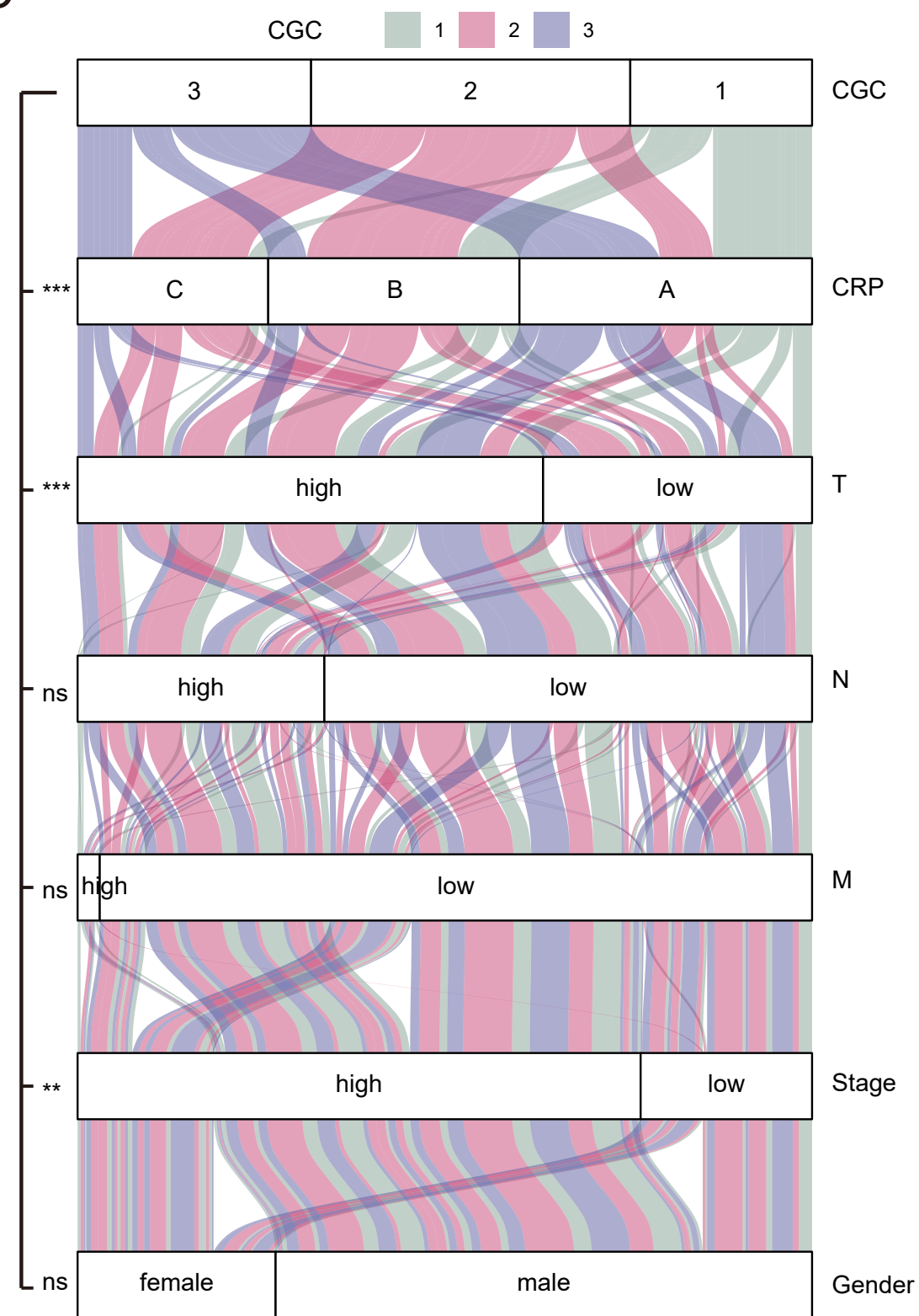

D

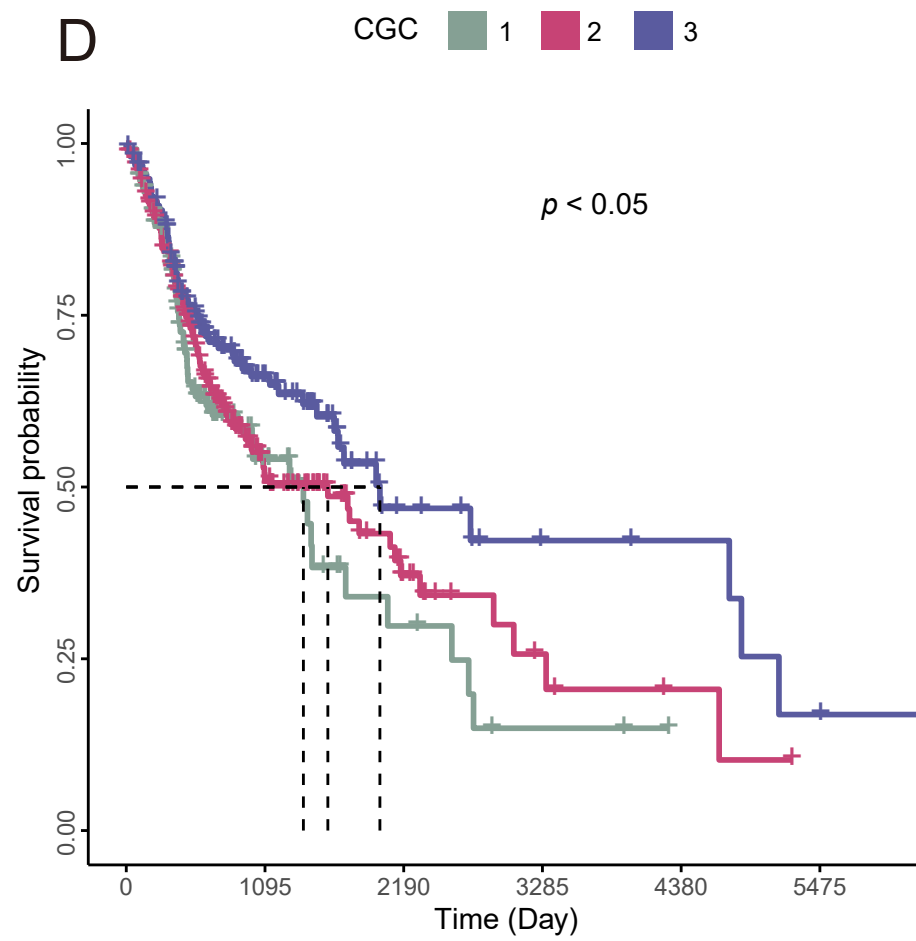

E

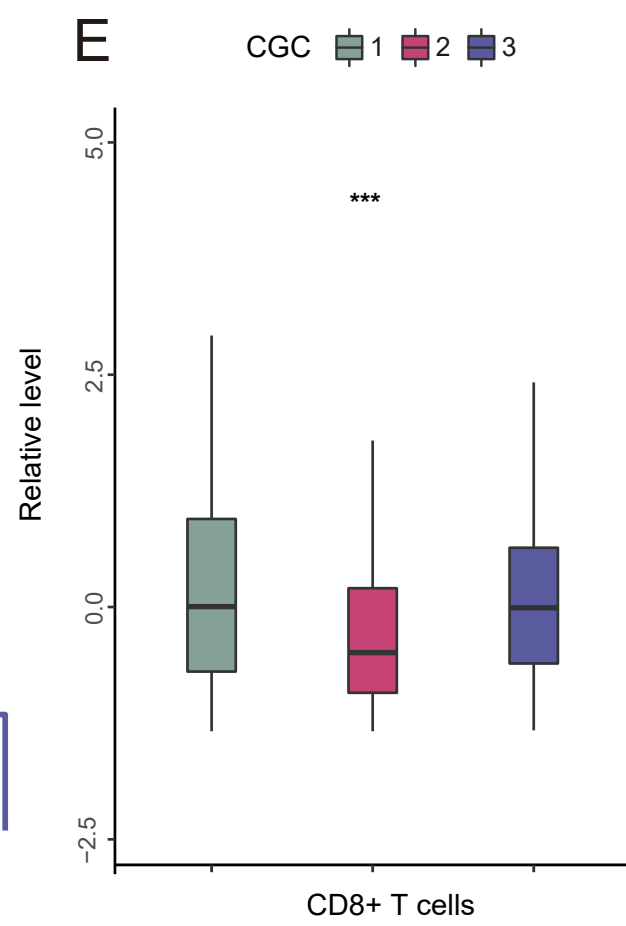

F

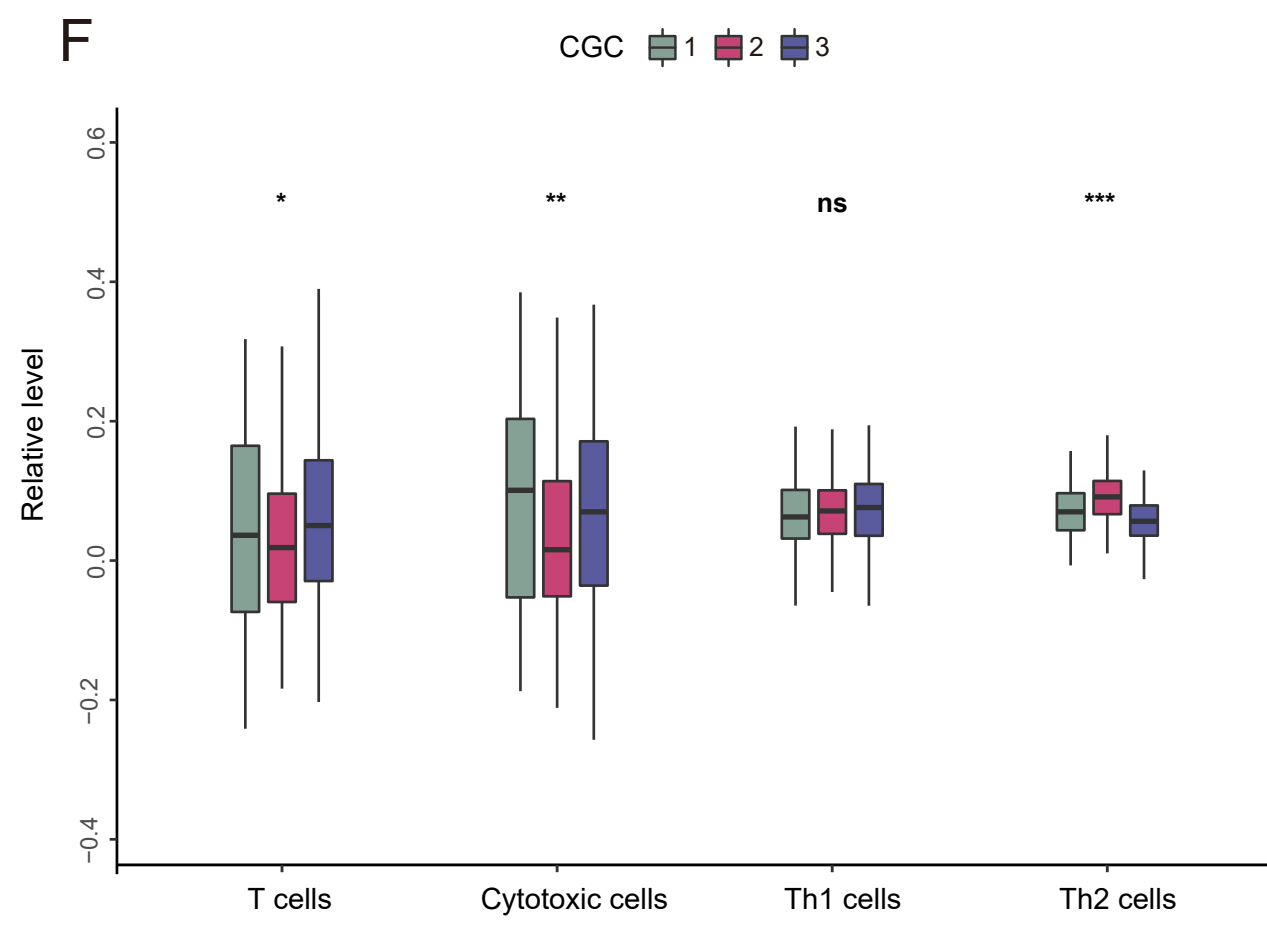

G

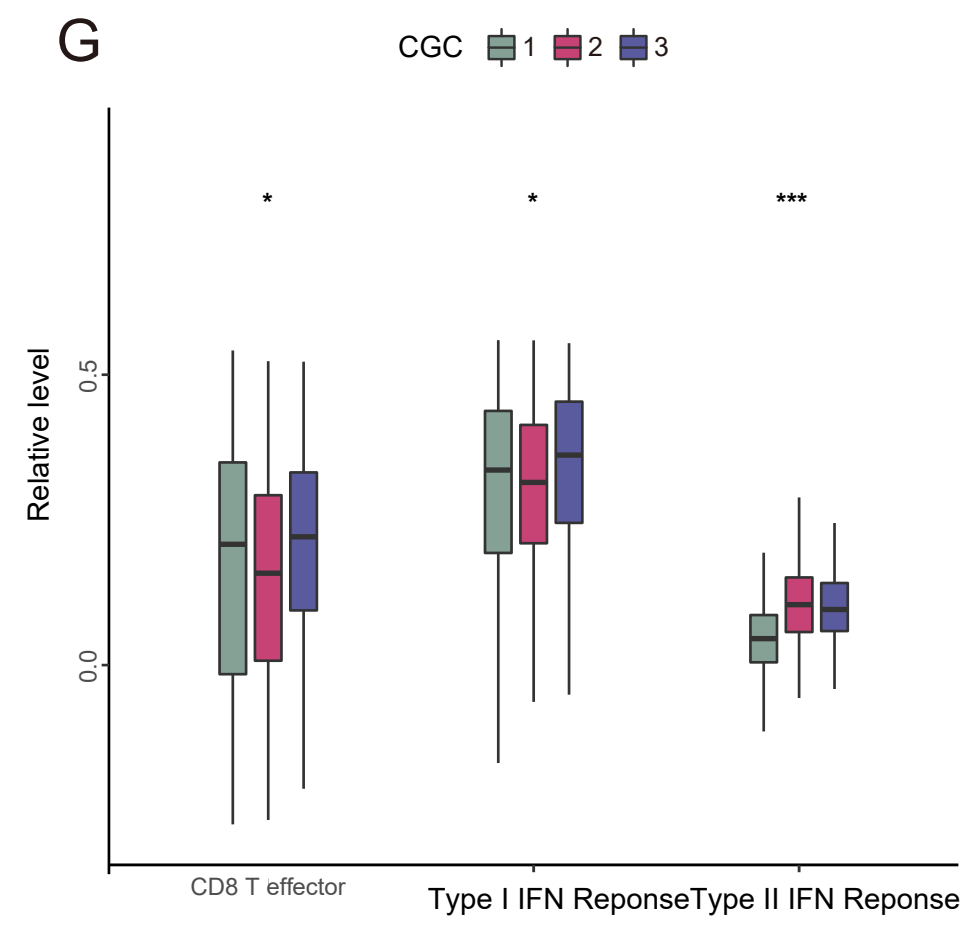

Supplement: Supplementary Materials — Figure 1: workflow of our study. The workflow of our study and analytical pipeline. Figure 2: comparison of PER3 expression in different subgroups. Box plots of PER3 expression in different T (A), N (B), M (C), stage (D), CRP subtypes (E), and CGC subtypes (F). Figure 3: CGC exhibited different TME and prognosis. (A) Consensus clustering matrix of HNSCC patients based on CSGs (k = 3). (B) Expression level of CSGs in the TCGA cohort. (C) A Sankey diagram exhibiting the relationship of CGCs, CRP and clinical features (T low = T1 + T2; T high = T3 + T4; N low = N0 + N1; N high = N2 + N3 + NX; M low = M0; M high = M1 + MX; grade low = G1 + G2; grade high = G3 + G4 + GX; stage low = I + II; and stage high = III + IV). (D) OS of different CGC subtypes. (E) A boxplot of CD8+ T cell abundance for three CGCs. (F) Difference in immune signatures among different CGCs. (G) GSVA analysis among different CGCs. Figure 4: validation of the performance of circadian score in different subgroups. Kaplan-Meier curves of OS based on circadian score in different T (A), N (B), M (C), stage (D), grade (E), and gender (F). [file 9946911.f1.zip › Sfigure3.pdf]

**A**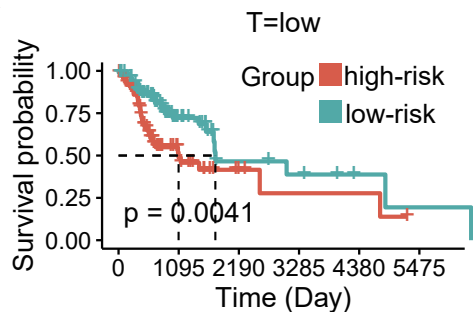**B**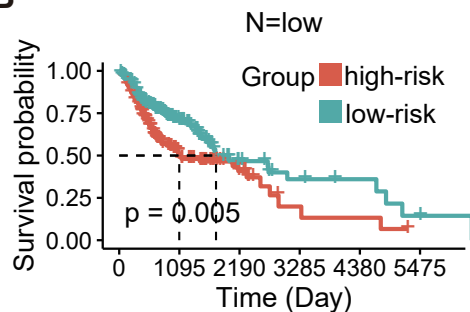**C**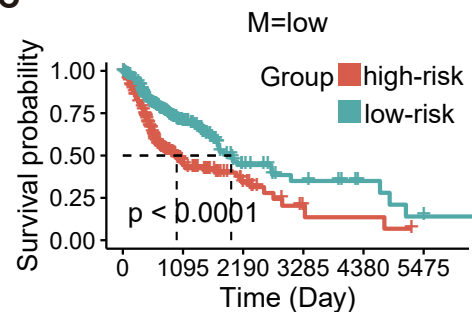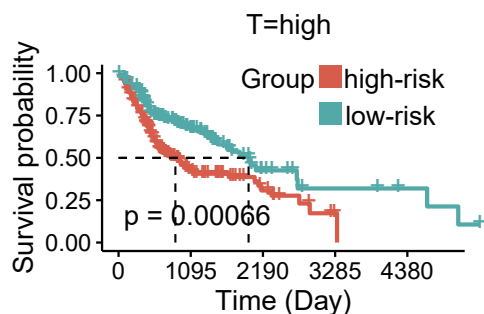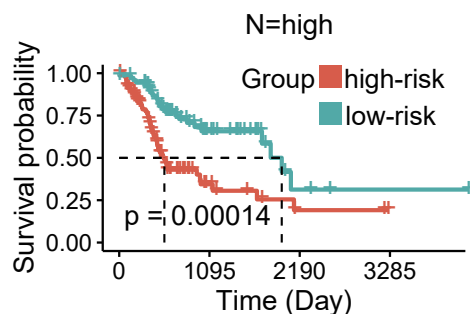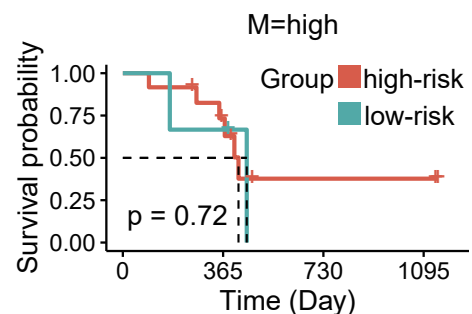**D**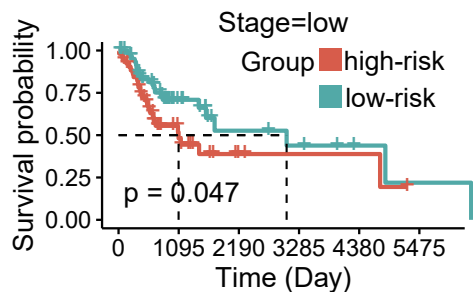**E**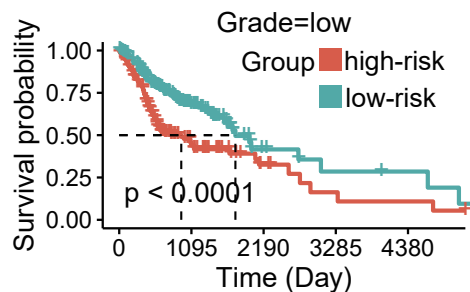**F**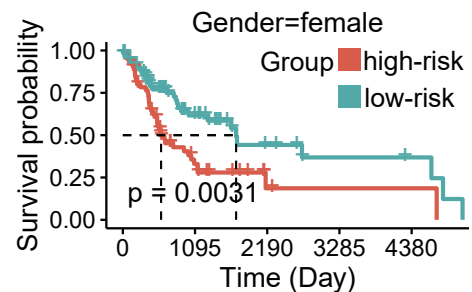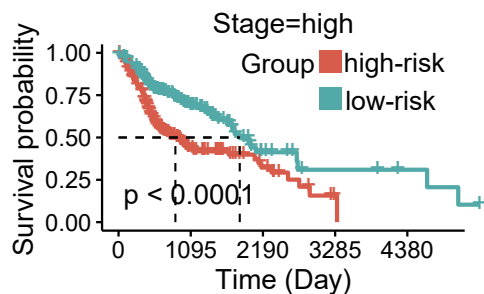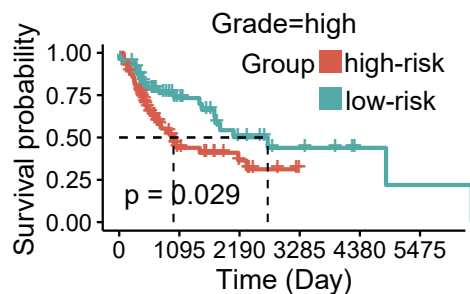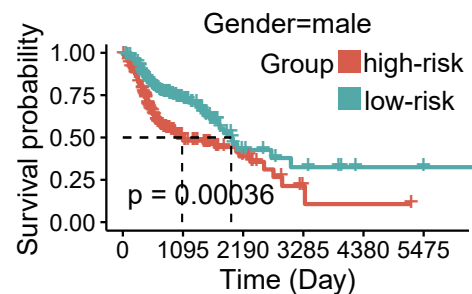

Supplement: Supplementary Materials — Figure 1: workflow of our study. The workflow of our study and analytical pipeline. Figure 2: comparison of PER3 expression in different subgroups. Box plots of PER3 expression in different T (A), N (B), M (C), stage (D), CRP subtypes (E), and CGC subtypes (F). Figure 3: CGC exhibited different TME and prognosis. (A) Consensus clustering matrix of HNSCC patients based on CSGs (k = 3). (B) Expression level of CSGs in the TCGA cohort. (C) A Sankey diagram exhibiting the relationship of CGCs, CRP and clinical features (T low = T1 + T2; T high = T3 + T4; N low = N0 + N1; N high = N2 + N3 + NX; M low = M0; M high = M1 + MX; grade low = G1 + G2; grade high = G3 + G4 + GX; stage low = I + II; and stage high = III + IV). (D) OS of different CGC subtypes. (E) A boxplot of CD8+ T cell abundance for three CGCs. (F) Difference in immune signatures among different CGCs. (G) GSVA analysis among different CGCs. Figure 4: validation of the performance of circadian score in different subgroups. Kaplan-Meier curves of OS based on circadian score in different T (A), N (B), M (C), stage (D), grade (E), and gender (F). [file 9946911.f1.zip › Sfigure4.pdf]
